# Editorial for the Special Issue on Advanced Thin Films: Design, Fabrication, and Applications, 2nd Edition

**DOI:** 10.3390/mi17040420

**Published:** 2026-03-30

**Authors:** Elena Kalinina

**Affiliations:** 1 Laboratory of Complex Electrophysic Investigations, Institute of Electrophysics, Ural Branch of the Russia Academy of Sciences, 620016 Yekaterinburg, Russia; jelen456@yandex.ru; Tel.: +7-343-267-87-82; 2 Department of Physical and Inorganic Chemistry, Institute of Natural Sciences and Mathematics, Ural Federal University, 620002 Yekaterinburg, Russia

The Special Issue “Advanced Thin Films: Design, Fabrication and Applications, 2nd Edition” was devoted to thin films as functional coatings in optoelectronics, electrochemical systems, adaptive microsystem devices, biocompatible implants, and memory devices. The Special Issue contains nine articles that examine aspects of fabrication, testing, optimization of characteristics, and technology of devices based on functional thin films. The topics of the presented works are devoted to current areas of modern research, including such promising objects as polymer films, memristors, photoconductors, and LED diodes. The research was conducted using modern equipment for thin-film production and testing and was primarily of a technological nature.

Modification of the titanium surface by polymer coatings allows for the obtaining of new functional properties of this material for use in electrochemical devices. Chiara Frezza et al. (Contributor 1) investigated the poly(2-(dimethylamino)ethyl methacrylate) (PDMAEMA) polymer material as a surface modifier for titanium. This polymer is characterized by solubility in water and a change in properties depending on the pH value [1]. At low pH, protonation of the amino groups of the polymer occurs, which becomes hydrophilic, whereas at high pH, deprotonation of the amino groups occurs, and the polymer acquires hydrophobic properties. The properties of the PDMAEMA polymer include a coil-to-globule transition when heated above 30–50 °C, depending on the pH of the aqueous solution and the polymer concentration [2]. The pH-controlled properties of the PDMAEMA polymer coating are of interest for modifying surface properties and regulating ion transport in electrochemical devices as well as in molecular machines. The authors investigated approaches to implementing covalent attachment of PDMAEMA chains to titanium surfaces. The graft-to strategy involved the addition of polymer chains to titanium, while the graft-from strategy involved the synthesis of the polymer by polymerization of the monomer on immobilized active centers on the titanium surface. The wettability properties of the polymer surface were investigated, the SEM study of the coating morphology was carried out using Fourier-Transform Infrared Spectroscopy (FTIR), and electrochemical properties were determined by impedance spectroscopy in the Na_2_SO_4_ electrolyte solution; an increase in the corrosion resistance of titanium with a polymer coating was shown. The authors suggest the applicability of their results for integration into adaptive microsystem devices with adjustable properties.

The development of proton exchange membrane fuel cells (PEMFCs) is a promising direction for the development of low-temperature electrochemical energy generation devices [3]. The authors considered the use of thin-film thermocouples as temperature sensors to solve the problem of monitoring and diagnosing the internal temperature state of PEMFCs in order to increase their durability. The use of thin-film thermocouples (TFTCs) opens up new possibilities due to their ability to be integrated into a design, low thermal inertia, small size, and accuracy of temperature measurements. Huijin Guo et al. (Contributor 2) investigated Ni-Cr/Ni-Si (800 nm) thermocouples with a SiO_2_ protective layer formed by magnetron sputtering. The conducted research was aimed at determining the characteristics of the dynamic process of heat transfer and establishing a temperature regime using thermocouples of different sizes. Additionally, the processes of degradation and destruction of coating—the occurrence of cracks and peeling— were studied to determine the optimal size of thermocouples in terms of dynamic characteristics when measuring temperature and the durability of the thin-film structure.

The properties of ZrO_2_ films obtained by atomic layer deposition (ALD) were investigated. Magnesium alloys are being considered as an alternative to stainless steel and titanium alloys for use as implants [4]. However, a disadvantage of magnesium alloys is their reactivity in a biological environment [5]. Pi-Chen Lin et al. (Contributor 3) used equal-channel angular pressing (ECAP) in conjunction with the subsequent formation of a ZrO_2_ protective layer with a thickness of 18–19 nm using the ALD method. The implemented ECAP technology made it possible to improve the microstructure of the magnesium alloy and increase its corrosion resistance and the homogeneity of the distribution of secondary phases. On the other hand, the ZrO_2_ film resulted in the protection of the metal surface from environmental influences and had biocompatibility properties, and the use of ECAP treatment of the Mg-Ca alloy also contributed to the improvement of the corrosion resistance of samples with a protective ZrO_2_ film. Previously, the possibilities of using ZrO_2_ as a protective coating were also presented in work [6].

Thin films of metal oxides have a number of optical and electrical properties that are promising for use in modern optoelectronic devices, including photodetectors, infrared sensors, and solar cells [7]. The well-known semiconductor material SiC is used in power electronics devices. A recent review by Michail Gavalas et al. was devoted to the analysis of theoretical and experimental investigation of the formation of polycrystalline SiC by the low-pressure chemical vapor deposition [8]. A relatively new promising material for optoelectronic devices is NiV_2_O_6_, films of which have the necessary set of optical properties for creating photodetectors [9]. Ahmed Kotbi et al. (Contributor 4) investigated the properties and characteristics of the formation of NiV_2_O_6_ thin films by the Nebulizer Spray Pyrolysis method for use in a photodetector. The optical and structural features of the obtained films were determined depending on their thickness and porosity; in particular, such properties as the optical band gap (Eg), absorption coefficient (α), extinction coefficient (k), and refractive index (n) were investigated. NiV_2_O_6_ films were deposited onto glass substrates heated to 250 °C. The obtained NiV_2_O_6_ films were 0.86–2.94 µm thick and exhibited a well-developed pore system. The possibility of obtaining films sensitive to optical radiation using inexpensive spray pyrolysis technology was demonstrated. This creates the possibility of applying the obtained results to a wide range of optoelectronic devices.

The development of new principles for data storage and storage devices is necessary from the point of view of the development of information systems for processing big data and artificial intelligence. The creation of memristor-based storage devices offers the potential to overcome the existing limitations of storage devices in data processing systems; additional capabilities may arise from using memristors not only for storage but also for data processing, including within neural networks [10,11]. Yulin Liu et al. (Contributor 5) investigated devices based on Au/HfO_2_/Pt (S1), Au/Ta_2_O_5_/Pt (S2), and Au/Ta_2_O_5_/HfO_2_/Ta/Pt(S3) layers. The characteristics of single-layer (S1, S2) and bilayer structures (S3) were compared in terms of stability, switching voltage, and state regulation. The authors interpret the differences in the properties of the studied memristor samples based on the concept of changes in Gibbs free energy and the formation and migration of oxygen ion vacancies in oxide layers. According to the data obtained, memristor structures with a double oxide functional layer demonstrated the best performance. A physical model for improving device performance was proposed, making it possible to use resistive memory devices.

Micro-light-emitting diodes (micro-LEDs) have become widely used due to their properties such as high brightness, durability, small size, and high resolution of LED arrays (high pixel-per-inch—PPI values) when used in displays [12]. In a recent review, circuit design solutions for active matrix displays were considered [13]. Creating high-resolution displays is an important task to achieve maximum visual comfort [14]. In this regard, research into the formation of indium tin oxide (ITO) layers for creating electrical contacts to the functional layers of LED diodes, such as p-type GaN, is of great importance. Such ITO layers must have high conductivity and transparency. Eun-Kyung Chu et al. (Contributor 6) studied micro-LED arrays with an InGaN/GaN core layer structure formed on a sapphire substrate. The production of the ITO layer for high-resolution micro-LED displays was optimized. The authors demonstrated the use of electron beam evaporation (e-beam) and sputtering technologies to form ITO layers on a sapphire substrate, achieving a resolution of 1692 PPI. The morphological features and microstructures of the obtained layers were studied, and the achieved resistivity values and emitting properties of blue micro-LED arrays were compared.

Luis Alberto Cantera et al. (Contributor 7) optimized the production technology of a photoconductor with a planar heterojunction architecture based on an organic semiconductor film. The use of organic materials with semiconducting properties opens the possibility of obtaining functional films for optoelectronic devices on various substrates, including flexible and non-planar substrates [15]. The functioning of a photoconductor is based on a change in its conductivity under the influence of radiation; in this regard, it is important to conduct research into the electrical properties of films and charge transfer mechanisms. In the work presented by Luis Alberto Cantera Cantera et al., the task was to fabricate and characterize an optical device based on organic layers of copper phthalocyanine (CuPc) and bathocuproine (BCP), which performed the function of transporting holes and electrons. The indium tin oxide (ITO) layer was used as the anode due to its conductive and transparent properties. Layered heterostructures were formed on a flexible polyethylene terephthalate (PET) substrate. A study was conducted on the response of the obtained structures to radiation, specifically, the change in J-V curves under various conditions, including changes in the radiation pattern and temperature. The authors demonstrated the applicability of the obtained heterostructures for the formation of flexible optoelectronic devices. Junjie He et al. investigated processes in thin-film waveguides based on lithium niobate, which is promising for the development of nonlinear optics devices [16].

Shimin Ge et al. (Contributor 8) investigated the properties of thin-film transistors (TFTs) obtained by the back-channel-etched (BCE) method based on amorphous InGaZnO (a-IGZO), which are widely used for the production of active matrix displays [17,18]. BCE technology is widely used in TFT fabrication due to its technological advantages. However, electrical instability of a-IGZO TFTs has been observed at elevated temperatures and humidity [19], as well as when thermal stress occurs. The manifestation of instability of a-IGZO structures is associated with an increase in the concentration of oxygen ion vacancies in the reverse channel. The work presented by Shimin Ge et al. is aimed at identifying the causes and mechanisms of the occurrence of a high density of oxygen ion vacancies in order to reduce the degradation processes of TFT structures based on thin a-IGZO films (80 nm) on a glass substrate obtained by magnetron sputtering technology. The changes in the sheet resistance of the IGZO film were determined under various conditions, as well as after etching to a specific depth to determine the change in resistance depending on the residual layer thickness, which allowed for an analysis of the distribution of oxygen defects. Based on these results, the authors developed a technology for removing the surface layer of the back channel with a high density of oxygen defects, improving the stability of TFT structures.

Sunhyun Park et al. (Contributor 9) demonstrated the feasibility of enhancing luminous efficiency by using a nanoscale vacuum photonic crystal (nVPCL) with a corrugated translucent electrode in an organic light-emitting diode (OLED). The approaches developed by the authors are promising in the field of development of optoelectronic energy conversion devices, for which the key parameter is luminous efficiency [20]. Increased luminous efficiency can be achieved by reducing losses due to reduced internal reflection. To address this issue, the authors proposed using a hollow microcavity electrode (HME) with an nVPCL layer and a periodic line structure. This solution improved OLED performance in terms of luminous efficiency and color purity, which holds promise for optoelectronics.

In conclusion, it should be noted that future research will be most productive when combining various thin-film formation technologies with theoretical methods of analysis and modeling, focusing on the relationships between the structural parameters and functional properties of new materials. Qualitatively new research impacts can be expected from an interdisciplinary approach and the integration of various functional elements—micromachines, optoelectronics, and electrochemical converters—into a single device.
